# Case report: Carcinoma en cuirasse—widespread cutaneous metastases from breast adenocarcinoma

**DOI:** 10.3389/fonc.2026.1742907

**Published:** 2026-03-04

**Authors:** Chongyi Wei, Jianping Long, Tao Yang, Haicun Zhou, Xiaoke Chai

**Affiliations:** Department of Breast Surgery, Gansu Provincial Maternity and Child-care Hospital/Gansu Provincial Central Hospital, LanZhou, China

**Keywords:** breast cancer, carcinoma en cuirasse, case report, clinical presentation, treatment

## Abstract

**Rationale:**

Cutaneous metastatic carcinoma is a malignant tumor that originates outside the skin. Studies have found that the most common primary tumor in female patients with cutaneous metastatic carcinoma is breast cancer. Cutaneous metastatic carcinoma spreads to the adjacent skin through blood vessels, lymphatics, or interstitial spaces. It forms ulcerative or nodular lesions. Carcinoma en cuirasse is characterized by extensive induration and sclerosis of the skin, resembling armor, and is a rare form of cutaneous metastatic carcinoma.

**Patient concerns:**

We report a rare case of a 59-year-old patient who developed dark red nodules on the skin of the breast in 2013. These nodules gradually enlarged, ultimately causing the breast to lose its normal shape. Subsequently, the skin on the chest and back thickened, accompanied by local erosion and ulceration that gave the skin a hardened, armor-like appearance.

**Diagnoses:**

Ultrasound and chest CT suggest diffuse infiltration in the subcutaneous fat of the neck and thorax. Core-needle biopsy pathology reveals infiltrating micropapillary carcinoma, with tumor invasion of the dermis, the tumor clearly originates from the breast.

**Interventions:**

The first-line treatment for advanced-stage breast cancer is the combination of cyclin-dependent kinase 4/6 inhibitors (CDK4/6 inhibitors) and aromatase inhibitors (AIs), specifically Abemaciclib and Anastrozole.

**Outcomes:**

The patient has been treated for 44 months, and the disease has significantly improved. This is evidenced by the absence of tumor tissue in the biopsy of the affected skin.

**Lessons:**

Breast cancer cutaneous metastasis, although rare, requires high vigilance, especially in the HER2-negative Luminal B subtype. The efficacy data on CDK4/6 inhibitors combined with endocrine therapy as a standard regimen for cutaneous metastasis is still lacking. It is necessary to enhance mechanistic studies and clinical observations, taking molecular characteristics into account.

## 1 Introduction

For Breast cancer ranks as the most prevalent malignant tumor among women and is notably the solid tumor most frequently associated with cutaneous metastasis. The incidence of skin metastasis across all metastatic cancers ranges from 0.7% to 6.4% (1, 2), with breast cancer accounting for approximately 30% of these instances (3). Cutaneous metastatic cancer refers to the metastasis of malignancies originating from outside the skin to the skin, in which tumor cells spread to the skin through blood vessels, lymphatic ducts, or interstitial spaces, forming ulcerative or nodular lesions. One rare form is “Carcinoma en Cuirasse,” a type of cutaneous metastatic cancer (4), characterized by widespread induration and sclerosis of the skin, resembling armor. Cutaneous metastasis often indicates that the disease has reached an advanced stage, and its pathogenesis involves multiple factors. Therefore, understanding the characteristics and patterns of cutaneous metastasis of breast cancer and exploring the factors affecting the timing of skin metastasis and survival are of practical clinical significance. Additionally, summarizing appropriate and effective treatment methods and diagnostic strategies is important. The author encountered a case of extensive cutaneous metastasis of breast cancer in clinical practice, which is described below.

## 2 Case presentation

**Figure 1:**
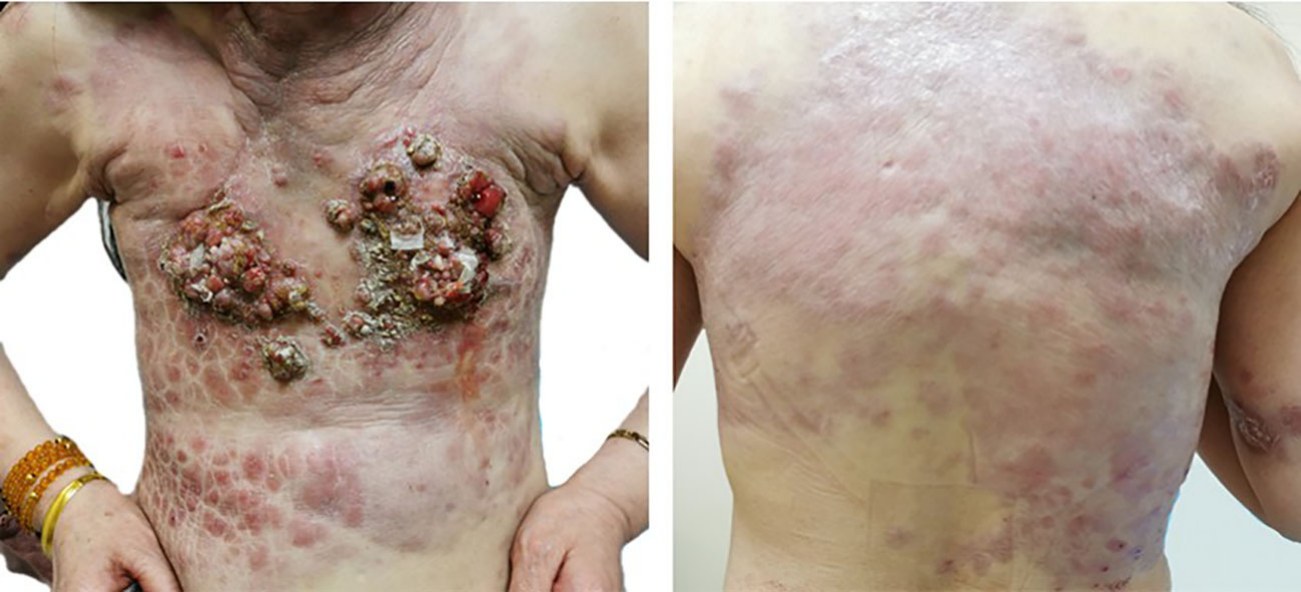
The patient’s condition upon initial consultation. A 59-year-old Asian woman first presented in 2022, with both breasts showing abnormal shape, characterized by local erosion and ulceration. Numerous irregular, red papules were also observed on the neck, chest, back, and right upper arm.

**Figure 2:**
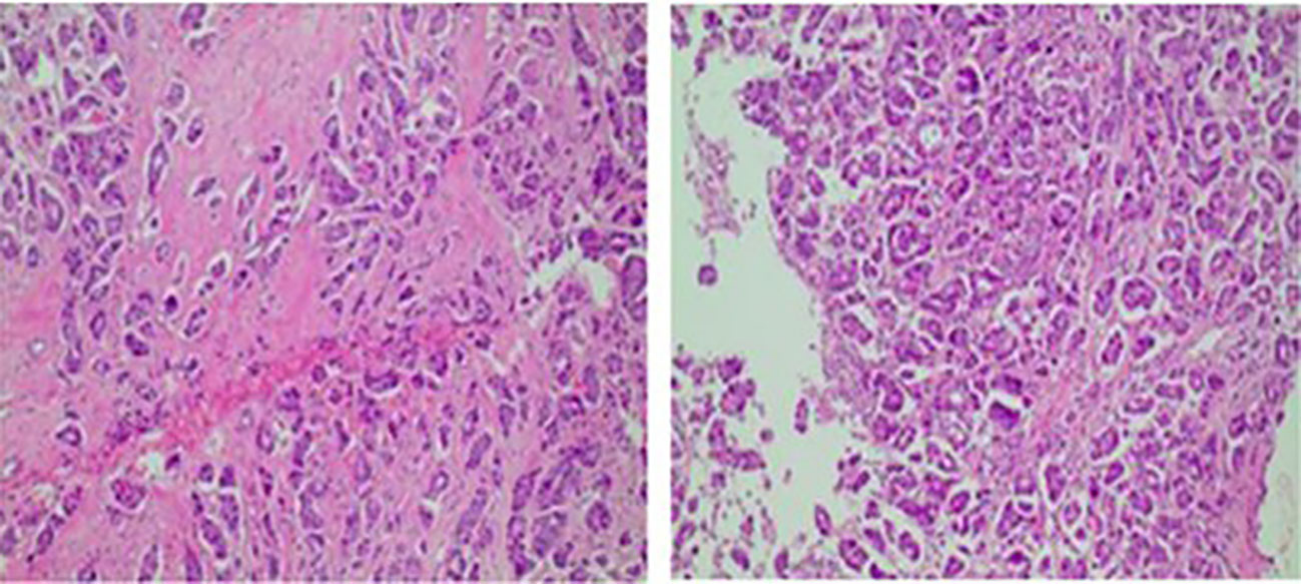
Core-needle biopsy pathology, consistent with infiltrative micropapillary components. Core needle biopsy pathology shows tumor cells were arranged linearly, with dense fibrosis in the surrounding tissue, decreased vascularity, and the presence of micropapillary infiltrative components within the tumor nests. The tumor invades the epidermis, showing positive vascular and lymphatic invasion.

**Figure 3:**
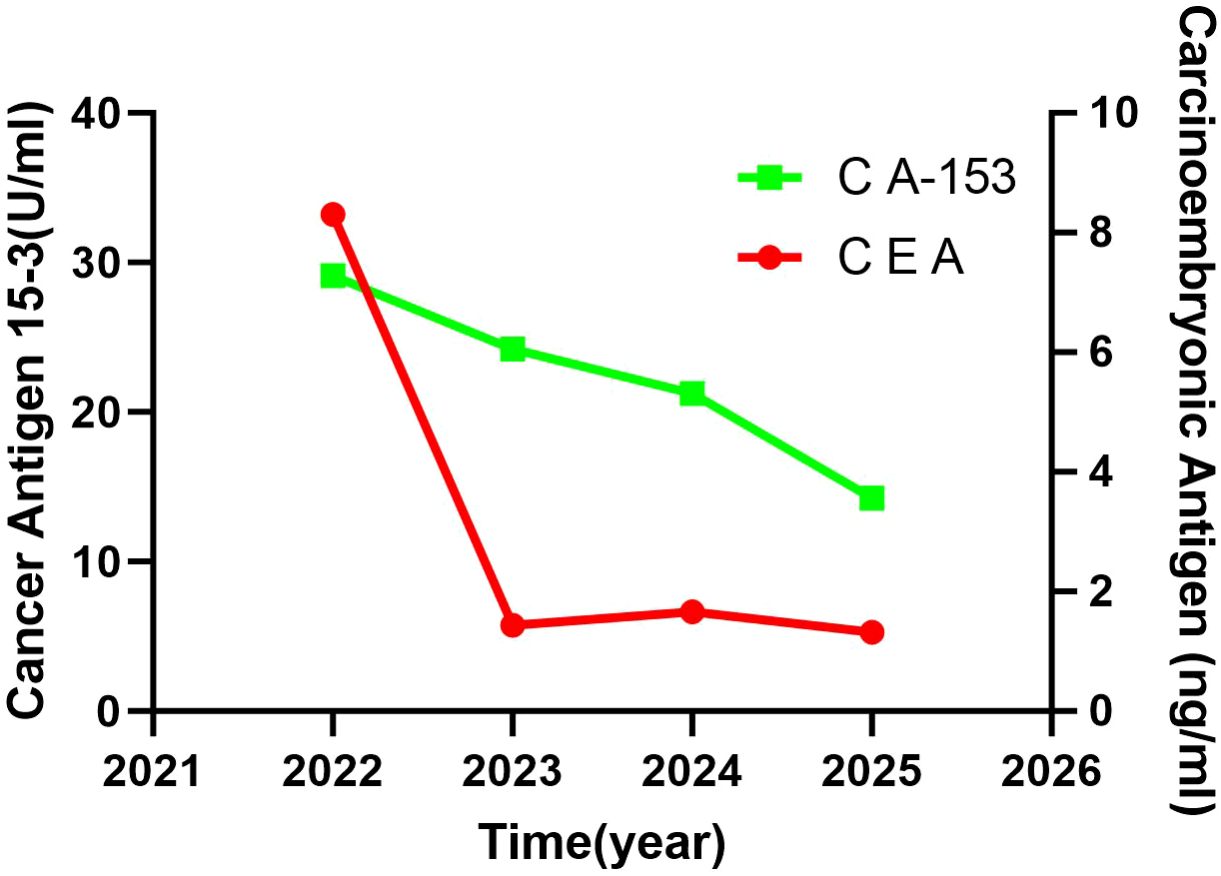
Tumor marker change curve(from 2022 to 2025). From 2022 to 2025, patient treated with first-line CDK4/6 inhibitors, combined with aromatase inhibitors, the tumor markers CEA and CA-153 showed a significant decrease.

**Figure 4:**
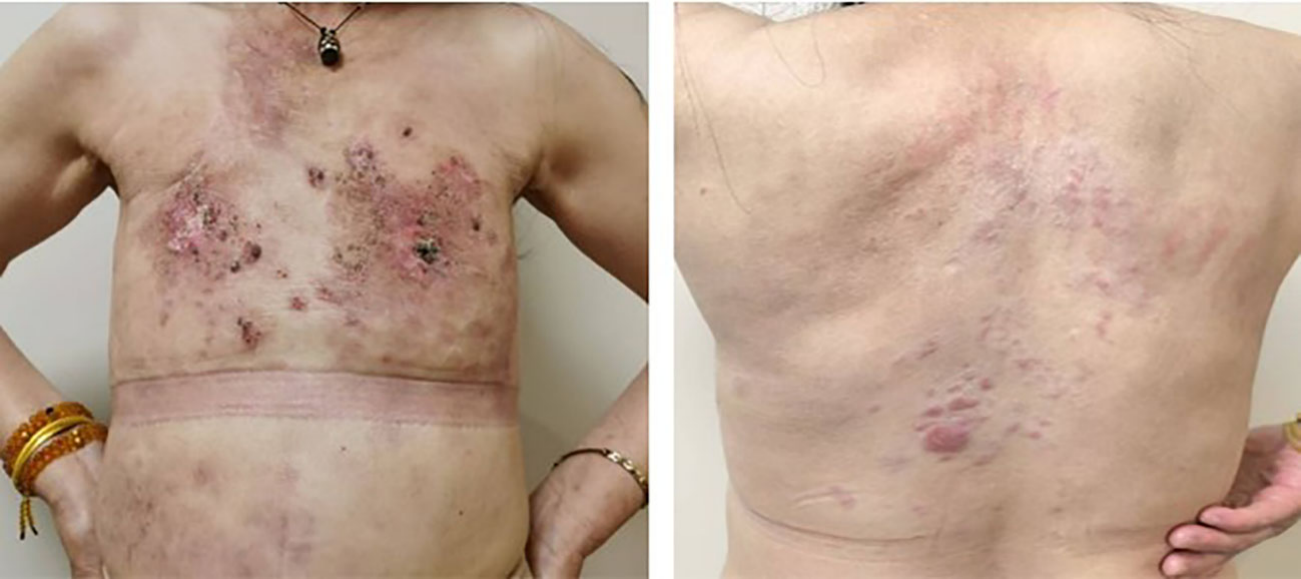
The patient’s condition in July 2022. After 4 months of treatment, the patient’s skin symptoms at the affected site have significantly improved, and the disease status has been controlled. A second biopsy is then performed to further assess the disease status.

**Figure 5:**
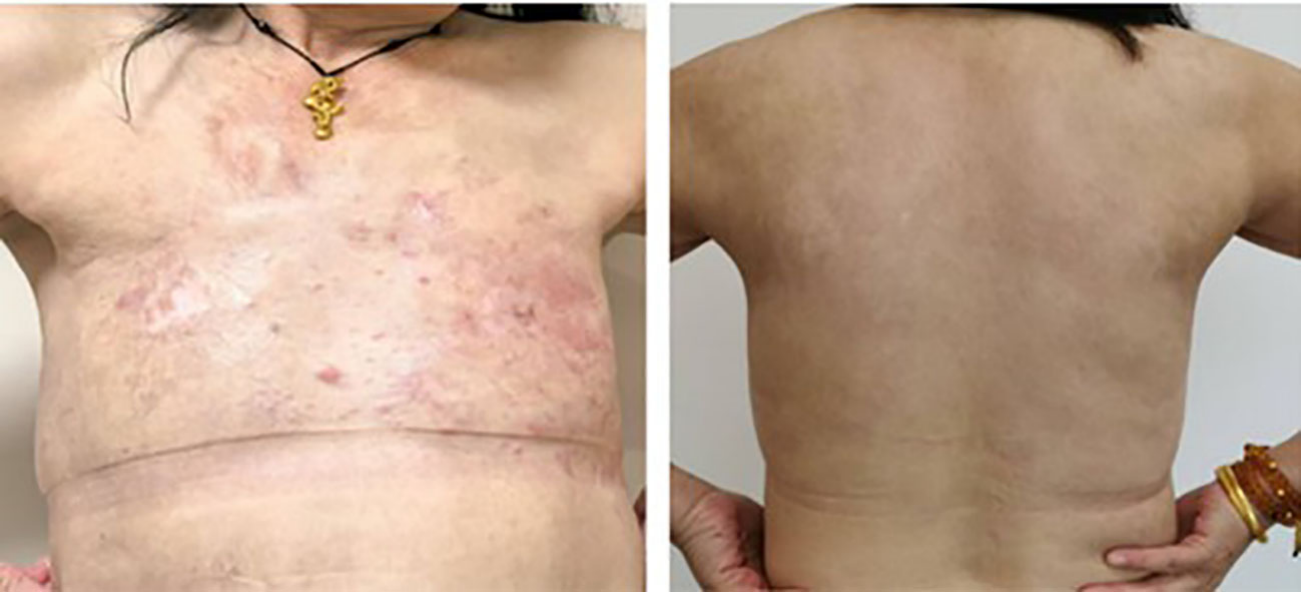
The patient’s condition in May 2024. After 26 months of treatment, the cutaneous nodules on the patient’s chest and back have largely resolved and the clinical condition has further improved.

The patient in this case is a 59-year-old Asian woman who is naturally postmenopausal, with no family history of tumors. In 2013, she developed red nodules on the skin of both breasts, which progressively increased in size and extended to the anterior chest and back, occasionally accompanied by discomfort. No treatment was initiated at that time. In February 2022, she sought medical attention, and a physical examination revealed deformities in both breasts, characterized by local erosion and ulceration. Numerous irregular, red papules were also observed on the neck, chest, back, and right upper arm (**Figure 1**). On February 21, 2022, the patient underwent a core needle biopsy. The pathological examination showed tumor cells were arranged linearly, with dense fibrosis in the surrounding tissue, decreased vascularity, and the presence of micropapillary infiltrative components within the tumor nests (**Figure 2**). The diagnosis confirmed infiltrative micropapillary carcinoma, with evidence of tumor invasion into the epidermis. Both vascular and lymphatic duct invasion were positive. Molecular characterization classified the tumor as Luminal B subtype, exhibiting strong positivity for estrogen receptor (ER) at 90%, moderate positivity for progesterone receptor (PR) at 40%, HER2 2+ (FISH negative), and a Ki-67 index of 80%. Imaging studies indicated diffuse exudation in the subcutaneous fat of the neck and chest, with no evidence of visceral metastasis; tumor markers were noted as follows: CEA at 8.3 ng/mL (normal <3.4), and CA-153 at 291 U/mL (normal <25) (**Figure 3**). A definitive diagnosis of advanced breast cancer with cutaneous metastasis was confirmed. In accordance with the breast cancer clinical practice guidelines, first-line therapy for this advanced-stage disease was commenced in March 2022. The treatment regimen included a combination of CDK4/6 inhibitors and aromatase inhibitors (Abemaciclib and Anastrozole). Treatment efficacy was assessed every two months based on the Response Evaluation Criteria in Solid Tumors 1.1; the evaluation result was Partial Response (PR). To further evaluate the disease status, biopsies of the chest wall tumor were performed at two distinct time points: four months after treatment initiation (July 2022, **Figure 4**) and twenty-six months later (May 2024, **Figure 5**). Pathological examination of the chest wall lesions revealed no evidence of residual tumor tissue. The patient has been under follow-up for 46 months since the start of therapy, during which significant alleviation of local symptoms has been observed, and no notable adverse events have been reported. This case report has received approval from the institutional ethics committee, and the patient has provided written informed consent for publication.

## 3 Discussion

Skin lesions resulting from breast cancer metastasis are typically characterized as multiple, firm, dark-colored, and painless nodules. In cases of extensive metastatic breast cancer spreading to the skin, the lesions may mimic cellulitis or exhibit an armored breastplate appearance (5, 6). Survival time following breast cancer skin metastasis is influenced by various factors. Some studies report a median survival time of 23–42 months after skin metastasis occurs (7, 8). Hu et al. demonstrated that the average survival time for patients with breast cancer skin metastasis combined with visceral metastasis is only 25 months (9).This case presents a Luminal B type (HER-2 negative) advanced breast cancer, characterized by the highly invasive and disfiguring skin metastasis known as carcinoma en cuirasse, which achieved significant efficacy after first-line treatment with AI combined with CDK4/6 inhibitors. This not only challenges the traditional perception of poor prognosis associated with widespread skin metastasis in breast cancer but also provides important innovative insights into precise treatment and prognosis assessment for advanced breast cancer from both clinical practice and mechanistic perspectives. The “ carcinoma en cuirasse “ is far from a simple sign of advanced disease, it reflects the tumor cells altering the skin microenvironment through six stages: local invasion, intravasation, survival in the circulation, arrest in a distant organ, extravasation, and micrometastasis formation and metastatic colonization. At the same time, it drives active fibrosis, leading to abnormal hardening and thickening of the dermis (10). Pablo Perez-Pinera attribute the pathophysiological basis of this phenomenon to pleiotrophin (PTN), which, through sustained activation of the PTN/RPTPβ/ζ signaling pathway, not only promotes the malignant phenotypic transformation of breast cancer cells but also remodels the tumor microenvironment by secreting PTN, including activating stromal fibroblasts, stimulating angiogenesis, and extracellular matrix synthesis, ultimately driving breast cancer progression to an invasive hard cancer phenotype (11). Other studies have shown that skin metastases are significantly enriched with pro-lymphangiogenesis genes and genomic instability drivers, leading to blockage and fibrosis of dermal lymphatic vessels by tumor cells. Additionally, frequent mutations in TP53 and deletions in STK11 accelerate the clonal evolution and metabolic adaptation of tumor cells within the skin microenvironment, collectively triggering characteristic skin sclerosis (12). Notably, some viewpoints suggest that this intense fibrotic response may be a failed attempt by the body to “trap” the tumor, inadvertently providing a physical barrier and biochemical microenvironment that promotes tumor cell survival, proliferation, and resistance (13). Although the local lesions presented strong invasiveness at initial treatment, the favorable treatment response indicates that the fundamental driving mechanism of tumor cell proliferation lies in its molecular phenotype, HR+ and HER2-negative status. Therefore, in alignment with the principles of precision medicine, the first-line standard treatment protocol is established based on pivotal phase III clinical trial data, including studies such as PALOMA-2, MONALEESA-2, and MONARCH-3. This regimen involves the combination of aromatase inhibitors with CDK4/6 inhibitors, which facilitates synergistic dual-targeted inhibition of the estrogen signaling pathway and suppression of cell cycle progression. Thereby slowing the advancement of the pathological condition (14–16).This further proves that in advanced breast cancer, the biological behavior of the tumor takes precedence over the morphological presentation of its metastatic lesions. The “carcinoma en cuirasse,” as the initial manifestation of the disease, may represent a special tendency for local invasion. If its molecular subtype is responsive to systemic treatment (as in this case), the prognosis can be significantly better than traditional perceptions. Additionally, while systemic treatment controls the disease, local intervention is also key to improving quality of life. Local treatment methods, in addition to traditional surgery and radiotherapy, include techniques such as electrochemotherapy (ECT), photodynamic therapy (PDT), and topical therapy (TT), which are continuously developing in clinical practice. Research shows that the complete response rate for skin local lesions can reach 35.5%, and the objective response rate can reach 60.2% (17). While some advocate for sequential treatment after systemic therapy to reduce cumulative toxicity, there is also clinical experience supporting early synchronous intervention when symptoms are significant to quickly relieve patient suffering. Looking ahead, if the disease progresses, it is crucial to clarify the resistance mechanisms through re-biopsy. It is especially recommended to take multiple samples from fibrotic areas to obtain viable tumor cells. For example, novel oral SERDs can be used for ESR1 mutations, while combination therapy with PI3K inhibitors may be considered for PIK3CA mutations (18, 19). For cases with HER-2 low expression previously treated with CDK4/6 inhibitors, ADC drugs such as T-DXd represent an effective treatment option (20). In summary, given complex clinical manifestations, we must continue to adhere to precise diagnostic practices, apply evidence-based first-line regimens, and actively integrate treatment strategies to truly benefit patients.

## 4 Conclusion

Carcinoma en cuirasse is a highly aggressive cutaneous metastatic lesion of breast cancer, resulting from complex interactions between tumor cells and the skin microenvironment. This case demonstrates that, even with such extensive cutaneous metastasis, the overall prognosis still depends on the molecular phenotype of the tumor and whether visceral metastasis has occurred. Currently, the management of advanced breast cancer has entered an era of precision medicine, in which “biological behavior takes precedence over morphological features.” The successful management of this case validates the concept of precision medicine. It demonstrates that through early identification, evidence-based treatment, and dynamic evaluation, long-term effective control of the condition can be achieved, resulting in a favorable survival prognosis. In the future, continuous deepening of mechanism research and clinical data accumulation targeting specific metastatic sites will further enhance the overall treatment level, bringing more hope to patients.

## Data Availability

The original contributions presented in the study are included in the article/supplementary material. Further inquiries can be directed to the corresponding author.
